# Predictors of Safer Conception Practices Among HIV-Infected Women in Northern Nigeria

**DOI:** 10.15171/ijhpm.2019.27

**Published:** 2019-05-18

**Authors:** Zubairu Iliyasu, Hadiza S. Galadanci, Alfa I. Oladimeji, Musa Babashani, Auwalu U. Gajida, Muktar H. Aliyu

**Affiliations:** ^1^ Department of Community Medicine, Bayero University, Kano, Nigeria.; ^2^ Centre for Infectious Diseases Research, Bayero University, Kano, Nigeria.; ^3^ Department of Obstetrics and Gynecology, Bayero University, Kano, Nigeria.; ^4^ Department of Medicine, Bayero University, Kano, Nigeria.; ^5^ Department of Health Policy & Vanderbilt Institute for Global Health, Vanderbilt University Medical Center, Nashville, TN, USA.

**Keywords:** Safer Conception Practice, HIV/AIDS, Women, Northern Nigeria

## Abstract

**Background:** Persons living with HIV often face discrimination in safe sex and reproductive choices, especially in lowresource settings. This study assessed fertility desires and intentions, risk perception and correlates of ever use of at least one safer conception method among HIV-infected women attending a tertiary health facility in Kano, Nigeria.

**Methods:** Structured questionnaires were administered to a cross section of 328 of 427 eligible HIV-infected women. Fertility desires and intentions, risk perception and safer conception practice were analyzed. Logistic regression was employed to assess for predictors.

**Results:** Of the 328 respondents, 150 respondents (45.7%) wanted more children. The proportions of respondents aware of their transmission risk during pregnancy, delivery, and breastfeeding were 69.5%, 75.3%, and 78.9%, respectively. Further, 68.9% of respondents were aware of the prospects of bearing HIV-negative children without infecting their partners. About 64.8% of women were aware of at least one safer conception method. Safer conception methods everused by the participants include: antiretroviral therapy (ART) (36.7%), timed unprotected intercourse with (10.9%), and without pre-exposure prophylaxis (PrEP) (17.2%), intravaginal insemination (7.3%) and intrauterine insemination (4.7%). Safer conception practice was predicted by marital status (married versus single, adjusted odds ratio [AOR]=1.50, 95% CI =1.10-3.55), parity (2-4 versus 0, AOR=12.1, 95% CI=3.7-39.8), occupation (civil servants versus traders, AOR=0.37, 95% CI=0.16-0.86), husband’s serostatus (seroconcordant versus serodiscordant) (AOR=1.51, 95% CI=1.13-4.64), couple contraceptive use (users versus non-users) (AOR=1.62, 95% CI=1.16-5.83) and transmission risk perception (high risk versus low/no risk) (AOR=2.14, 95% CI=1.18-3.90).

**Conclusion:** We found high levels of fertility desires and intentions and moderate risk perception among a cohort of HIV-infected women in urban Kano, Nigeria. The use of safer conception practices was not common. Our findings underscore the need for healthcare provider capacity building to enhance safer conception counseling and service delivery.

## Introduction

The HIV/AIDS pandemic has challenged human development, especially in high burden low-resource settings.^1-4^ In 2016, women constituted nearly half (17.8 million) of the world’s 36.7 million people living with HIV.^5^ Of the 1.8 million new HIV acquisitions that year, 160 000 were in children. In Nigeria, an estimated 37 000 infants acquired HIV infection vertically in the same year, one of the highest globally.^5^ In sub-Saharan Africa, the virus is predominantly transmitted heterosexually. Childbearing among HIV-infected couples carries substantial risk of HIV transmission not only to the uninfected partner, but to the fetus as well. In addition, even among seroconcordant couples, there is the risk of acquiring a different strain through secondary transmission. Therefore, in the early phase of the epidemic, HIV-infected couples were not only discouraged from procreation, but offered termination of pregnancy to avert vertical transmission.^6^ However, these all changed with the discovery of reduced transmission risk through timed unprotected sexual intercourse, antiretroviral therapy (ART) pre-exposure prophylaxis (PrEP), artificial insemination and male medical circumcision.^7^ Recent additions include: sperm washing, intrauterine insemination, in-vitro fertilization and intra-cytoplasmic sperm injection.^8^ However, these methods are not 100% effective nor are they within reach of most affected couples in low-resource settings. Paradoxically, this is where fertility demand is high.^9^ It is therefore, likely that HIV-infected couples in these settings are conceiving naturally, despite well-documented risks.^10^

With increasing access to antiretrovirals and prevention of mother to child transmission services, the proportion of HIV-infected women in seroconcordant and serodiscordant partnerships who desire to have children has increased from about 26% to 69% in different regions.^11^ Recent studies of pregnant women on highly active antiretroviral treatment suggest that pregnancy does not speed up the progression of HIV disease.^12,13^ Despite these promising data, women living with HIV still face significant social and health system challenges when contemplating parenthood, including stigma and resistance from some healthcare providers and the general public.^14^ The current reach of affordable, timely and stigma-free safer conception methods is restricted. Although higher rates of use of safer conception methods have been reported in recent demonstration projects among HIV-positive women,^15^ the proportion of women practicing safer conception ranges from almost none in South East Asia to 7% in parts of sub-Saharan Africa.^16^ With the expected increase in patient enrolment on ART following the adoption of “test and treat” policy across the globe, even less time is likely to be available for discussions about reproductive plans and safer conception, particularly in low-resource settings.^17,18^

Very little is known about risk perception, fertility desires and intentions, safer conception practices and their predictors among HIV-infected women in northern Nigeria, the region with the highest total fertility rate in the country.^19^ The objective of this study is to assess the fertility desires and intentions, risk perceptions and predictors of ever use of at least one safer conception method among HIV-infected women attending a teaching hospital in northern Nigeria.

## Methods

### Study Setting and Population

The study was conducted at the S. S. Wali ART Centre, Aminu Kano Teaching Hospital (AKTH), Kano, Nigeria. AKTH is a 550 bed government-owned tertiary health institution serving a catchment population of over 13 million.^20^ The inhabitants are mainly Hausa-Fulani. The ART clinic operates daily, and does not charge for clinical examinations, laboratory tests and antiretroviral drugs. Patient support groups, counseling, testing and home-based services are also available. HIV infection is diagnosed using a sequential HIV rapid screening algorithm based on the national guidelines.^21^ All patients undergo pre-and post-test counseling.^22^ Included in the study were consenting women age 18-49 years, diagnosed with HIV (≥6 months earlier) attending AKTH. Persons who withheld consent or were too sick to be interviewed were excluded.

### Study Design and Sampling

We employed a cross-sectional study design. Sample size *n* was obtained using Fisher formula [n=z^2^p(1-p)/d^2^=, where z = standard normal variate (1.96), p = proportion of women aware of (≥1) safer conception method from a previous study (53%)^23^ and d = tolerable error of 5%]. The sample size obtained (n = 384) was then increased by 10% to account for anticipated non-response (384/0.90 = 427), giving a final sample size of 427.

Systematic sampling was used to recruit participants as they arrived at the ART center. Using average clinic attendance and estimated sample size, a sampling interval was computed. On arrival, the client was registered, given a sequential serial number, and allocated a consulting room. While waiting, clients were informed about the research. The first respondent was determined by picking a random number between 1 and the sampling interval. Subsequent respondents were identified by adding the sampling interval to the preceding respondent’s serial number. Women whose serial number tallied with the sampling process were individually invited into a separate room after the clinic consultation and informed consent obtained. Participant names were not recorded on the questionnaire and all other identifying information were kept confidential. Non-participation had no effect on subsequent care. Referral for professional counselling was available, if required. Only participants who provided informed consent were interviewed in private.

### Study Instrument and Data Collection

A pre-tested structured interviewer-administered questionnaire with five sections was adapted from a previous study.^24^ This previous tool provided the concepts, but the questions used in the present study were adapted for our population and re-validated. The first section inquired about socio-demographic characteristics. The second section elicited detailed obstetric history, HIV diagnosis, partner serostatus, disclosure and treatment history. Section 3 assessed transmission risk perception, fertility desires and intentions, while section four focused on awareness and practice of safer conception methods. Fertility desire was assessed using the question “Do you wish to have any/more children?” with 3 options, ‘Yes,’ ‘No,’ and ‘Undecided,’ whereas fertility intention was elicited using the question “Do you plan to have another baby within the next 3 years?” with ‘Yes’ and ‘No’ options. Awareness of safer conception methods was determined with the question “Are you aware of any safer conception method for HIV-positive couples?” This question was followed by “If yes, describe the method (s) you are aware of.” The interviewer was instructed not to read out the options, but to compare the description by the respondent with a list of options provided, and select all responses mentioned. Risk perception was assessed based on the response to questions on risk of infecting a partner during conception and the chances of serodiscordant and seroconcordant couples bearing an uninfected child. Other questions included risk of HIV transmission during pregnancy, childbirth and breastfeeding and whether or not HIV transmission via these routes can be prevented.

### Theoretical Framework and Measurements

Based on Miller’s Traits-Desires-Intentions-Behavior framework for understanding childbearing motivations,^25^ fertility desire was assessed by asking “Do you wish to have any (more) children?” with dichotomous response options “Yes” coded as 1 or “No” coded as 0. If the respondent answered “Yes,” they were asked the total number of children they desire to have. The response was recorded as a discrete number. Further, to assess fertility intention, respondents were asked, “Are you planning to have any (more) children in the next 3 years?” (“Yes” coded as 1 or “No” coded as 0). Practice of safer conception was classified as ‘Yes’ coded as 1, if the respondent ever practiced at least one method of safer conception, specifically one, a combination or all of the following: used antiretroviral drugs while trying to conceive, timed unprotected intercourse, timed intercourse with PrEP for HIV-negative partner, sperm washing with intrauterine insemination and artificial intra-vaginal insemination.

### Data Analysis

Data were analyzed using SPSS version 22.^26^ Continuous data were summarized using mean ± SD or median and range. Categorical data were presented as frequencies and percentages. At bivariate level, Pearson’s chi-square or Fisher’s exact test was used to assess significance of associations at *P *<* *.05. Risk perception was dichotomized into high and low/no risk perception based on the responses as follows: (1) Respondents who indicated there is no risk of infecting a partner during conception and no transmission risk in utero, at delivery and through breastfeeding were considered to perceive no risk; (2) Participants who were of the view that transmission was possible at least during one of these times, but was not preventable, were characterized as having low risk perception; (3) Respondents who felt that a transmission risk exists at all of these times, but that transmission is preventable, were considered as having high risk perception. A logistic regression model was developed with variables that had *P *<* *.10 at bivariate level or those that were considered conceptually important, irrespective of their significance at that level (age group, marital status, occupation, number of children, risk perception, fertility desire and couple contraceptive use, partner’s HIV status). The dependent variable was ‘ever practiced at least one safer conception method,’ classified as ‘Yes’ (coded as 1), if the respondent has ever practiced any, a combination or all of the following safer conception methods: used ART drugs while trying to conceive, timed intercourse with PrEP for HIV-negative partner, and artificial intra-vaginal insemination. Adjusted odds ratios (AORs) and associated 95% CI were used to compute effect estimates.

A secondary analysis of the same outcome was conducted only on women who desired children. In the second model, all the previous independent variables were included in the model, except fertility desire.

## Results

### Sociodemographic Characteristics

Of 427 eligible women, 328 completed the interviews, a response rate of 76.8%. Non-response was mainly related to earlier commitments. Respondents’ mean age (±SD) was 33.0 (±7.5) years. Over half of respondents were Hausa/Fulani (52.7%) and Muslims (60.7%). More than a quarter of respondents (29.0%) were teachers. Over two-thirds (71.7%) of respondents had at least secondary education, but nearly a fifth (19.2%) had no for­mal education (Table 1).

**Table 1:** Socio-Demographic and Obstetric Characteristics of HIV-Infected Women, Kano, Nigeria (N = 328)

**Characteristics**	**No. (%)**
Age group	
<30	104 (31.7)
30-39	139 (42.4)
≥40	85 (25.9)
Ethnicity	
Hausa/Fulani	173 (52.7)
Yoruba	53 (16.2)
Igbo	43 (13.1)
Others	59 (18.0)
Religion	
Islam	199 (60.7)
Christianity	129 (39.3)
Education	
No formal	63 (19.2)
Primary	30 (9.1)
Secondary	81 (24.7)
Post-secondary	154 (47.0)
Marital status	
Single	69 (21.0)
Married	195 (59.5)
Divorced	27 (8.2)
Widowed	37 (11.3)
Occupation	
Homemaker	47 (14.3)
Teaching	95 (29.0)
Trading	81 (24.7)
Civil servant	66 (20.1)
Others	39 (11.9)
Parity	
0	72 (22.0)
1	50 (15.2)
2-4	143 (43.6)
≥5	63 (19.2)

Majority of the respondents (59.5%) were married. The median parity of respondents was 2.0 (range, 0 to 10). Of the 259 ever married respondents, 167 (50.9%) had disclosed to their partners. Almost half (n = 122, 47.1%) of the ever married women had seroconcordant partners. The rest were either serodiscordant (n = 62, 23.9%) or partner status was unknown (n = 75, 29.0%).

### Risk Perception and Fertility Desires and Intentions

Table 2 presents risk perception, fertility desires and intentions. Over two-thirds of the women (68.9%) were aware of transmission risk during pregnancy (69.5%), delivery (75.3%) and breastfeeding (78.9%) in the absence of interventions. Over two-thirds (68.9%) of the participants were aware of the possibility of bearing HIV-free children without infecting their partners. Overall, (n = 122, 37.2%) respondents perceived the risk of HIV transmission to partner and foetus/infant as low or non-existent while (n = 206, 62.8%) perceived it as high.

**Table 2:** Risk Perception and Fertility Intention Among HIV-Infected Women, Kano, Nigeria, 2016 (N = 328 unless otherwise stated)

	**Yes** **No. (%)**	**No** **No. (%)**	**Do not Know** **No. (%)**
**Risk Perception**			
Possibility of serodiscordant couple conceiving without infecting the partner	226 (68.9)	33 (10.1)	69 (21.0)
Likelihood of serodiscordant couple bearing HIV-negative baby	243 (74.1)	21 (6.4)	64 (19.5)
Possibility of seroconcordant couple bearing HIV-negative baby	225 (68.6)	43 (13.1)	60 (18.3)
Risk of HIV transmission in utero	228 (69.5)	50 (15.2)	50 (15.2)
Risk of HIV transmission during delivery	247 (75.3)	31 (9.5)	50 (15.2)
Risk of HIV transmission through breastfeeding	262 (78.9)	24 (7.3)	42 (12.8)
Possible to prevent mother to child transmission of HIV?	206 (62.8)	122 (37.2)	-
**Fertility Desires and Intentions**			
Wants to have more children	150 (45.7)	147 (44.8)	31 (9.5)
Total number of (biological) children desired	‏-	-	-
None	22 (6.7)	-	-
1	16 (4.9)	-	-
2-4	143 (43.6)	-	-
≥5	147 (44.8)	-	-
Intents to have a child/another child in the next 3 years (n = 150)	134 (89.3)	16 (10.7)	-
Discussed fertility plans with healthcare provider (n = 134)	79 (59.0)	55 (41.0)	-
Healthcare provider supportive of fertility plans (n = 79)	75 (94.9)	4 (5.1)	-
Discussed fertility plans with husband/partner (n = 150)	118 (78.7)	32 (21.3)	-
Husband/partner supportive of fertility plans (n = 118)	108 (91.5)	10 (8.5)	-
Current contraceptive use	67 (20.4)	261 (79.6)	-
Partner's current contraceptive use	91 (27.7)	104 (31.7)	133 (40.6)

More than three-fourths of respondents who desire children had discussed their plans with their partners (78.7%). Most partners (91.5%) and healthcare workers (94.9%) were supportive. At the time of the study, 1 in 5 respondents (n = 67, 20.4%) and more than 1 in 4 partners (n = 91, 27.7%) were using contraceptives. In over half (n = 110, 56.4%) of the 195 currently married respondents, at least one partner was using a modern contraceptive method.

### Awareness and Practice of Safer Conception Methods

Table 3 presents awareness and ever-practice of safer conception methods. Almost two-thirds of women (n = 213, 64.9%) were aware of at least one method of safer conception. The most common methods of safer conception listed included ART (62.8%), timed unprotected intercourse (29.3%) and timed intercourse with PrEP for HIV-negative partner (23.2%). Sperm washing and artificial insemination were mentioned by less than 1 in 10 respondents. The proportions that ever practiced the listed methods of safer conception were 23.2%, 15.9%, 9.8%, 4.3%, and 6.7%, respectively.

**Table 3:** Awareness and Ever Practice of Safer Conception Methods Among HIV-Infected Women, Kano, Nigeria, 2016

	**Yes** **No. (%)**	**No** **No. (%)**
Awareness of safer conception methods (n = 328)		
Aware of at least one safer conception method for HIV-positive couples (n = 328)	213 (64.9)	115 (35.1)
Use of ART drugs	206 (62.8)	122 (37.2)
Timed unprotected intercourse	96 (29.3)	232 (70.7)
Timed intercourse with PrEP for HIV-negative partner	76 (23.2)	252 (76.8)
Sperm washing with intra uterine insemination	40 (12.2)	288 (87.8)
Artificial intra-vaginal insemination	34 (10.4)	294 (89.6)
Safer conception methods ever used (n = 328)		
ART drugs	76 (23.2)	252 (76.8)
Timed unprotected intercourse	52 (15.9)	276 (84.1)
Timed intercourse with PrEP	32 (9.8)	296 (90.2)
Sperm washing with intrauterine insemination	14 (4.3)	314 (95.7)
Artificial intra-vaginal insemination	22 (6.7)	306 (93.3)

Abbreviations: ART, antiretroviral therapy; PrEP, pre-exposure prophylaxis.

### Predictors of Safer Conception Practice

Table 4 presents the factors associated with practice of safer conception. At bivariate level, safer conception practice was associated with respondent’s age, ethnicity, religion, education, occupation, number of children, couple contraceptive use and risk perception (*P* < .05). At multivariate level, including all respondents, marital status, occupation, number of children, couple contraceptive use, husband/partner serostatus and risk perception remained statistically significant predictors of safer conception practice. Nearly half of the HIV-infected women (n = 150, 45.7%) interviewed wanted more children. The median number of children desired was 4 (range, 1 to 12). Most respondents (n = 134, 89.3%) intend to have a child within 3 years.

**Table 4:** Logistic Regression Model for Predictors of Safer Conception Practice Among HIV-Infected Women, Kano, Nigeria, 2016 (N = 328)

**Characteristics**	**Ever Practiced Safer Conception**	**Never Practiced Safer Conception**	**Crude Odds Ratio (95% CI)**	**AOR (95% CI)**	***P*** **Value**
Age group					
<30	36 (34.6)	68 (65.4)	Ref		
30-39	50 (36.0)	89 (64.0)	1.08 (0.73-1.92)	0.64 (0.41-1.41)	.38
≥40	44 (51.8)	41 (48.2)	2.08 (1.25-3.72)	1.39 (0.71-2.86)	.43
Marital status					
Single	14 (20.3)	55 (79.7)	Ref		
Married	93 (47.7)	102 (52.3)	3.46 (1.91-6.92)	1.50 (1.10-3.55)	.014^b^
Divorced	17 (63.0)	10 (37.0)	6.71 (2.64-17.81)	1.73 (0.48-5.34)	.66
Widowed	6 (16.2)	31 (83.8)	0.84 (0.33-2.31)	0.21 (0.07-0.78)	.031^b^
Occupation					
Trading	41 (50.6)	40 (49.4)	Ref		
Teacher	30 (31.6)	65 (68.4)	0.45 (0.24-0.83)	0.33 (0.15-0.72)	.034^b^
Civil servant	33 (50.0)	33 (50.0)	0.98 (0.51-0.77)	0.37 (0.16-0.86)	.027^b^
Others	26 (30.2)	60 (69.8)	0.42 (0.22-0.79)	0.44 (0.19-1.01)	.12
No. of children					
0	5 (6.9)	67 (93.1)	Ref		
1	29 (58.0)	21 (42.0)	18.50 (6.36-53.90)	26.2 (7.80-87.80)	.011^b^
2-4	68 (47.6)	75 (52.5)	14.10 (4.62-31.90)	12.1 (3.70-39.80)	.037^b^
≥5	28 (44.4)	35 (55.6)	10.70 (3.81-30.20)	5.60 (1.44-21.90)	.023^b^
Husband's serostatus^a^					
Serodiscordant	24 (38.7)	38 (61.3)	Ref		
Seroconcordant	71 (58.2)	51 (41.8)	2.24 (1.24-7.46)	1.51 (1.13-4.64)	.012^b^
Serostatus unknown	21 (28.0)	54 (72)	0.72 (0.17-0.93)	0.53 (0.22-0.83)	.024^b^
Couple contraceptive use^a^					
Yes	63 (61.2)	40 (38.8)	1.80 (1.47-7.59)	1.62 (1.16-5.83)	.032^b^
No	53 (34.0)	103 (66.0)	Ref		
Fertility desire					
No	60 (40.8)	87 (59.2)	Ref		
Yes	62 (41.3)	88 (58.7)	1.03 (0.78-3.64)	0.68 (0.12-2.65)	.67
Undecided	8 (25.8)	23 (74.2)	2.8 (0.53-7.45)	1.42 (0.25-5.63)	.53
Transmission risk perception					
Low/no risk	36 (29.5)	86 (70.5)	Ref		
High risk	94 (45.6)	112 (54.4)	2.61 (1.14-5.76)	2.14 (1.18-3.90)	.014^b^

Abbreviations: AOR, adjusted odds ratio; Ref, reference group. ^a^Only for ever married respondents. ^b^Significant at *P* < .05.

Specifically, after adjusting for other variables, married respondents had 50% increased chance of practicing safer conception relative to single women. Similarly, the likelihood of use of safer conception were 67% and 63% lower among teachers and civil servants, respectively, compared with traders. Further, women with one child had a much higher chance of using safer conception relative to nulliparous women.

Seroconcordant couples were 51% more likely to have practiced safer conception relative to serodiscordant couples. Similarly, women whose partner was of unknown serostatus were nearly half (47%) as likely to have practiced safer conception compared to serodiscordant couples. Couples using modern contraception were 62% more likely to practice safer conception compared to those who did not. Finally, respondents who perceived the risk of HIV transmission to infants and partners as high during conception had more than two-fold odds of practicing safer conception compared to those who felt the risk was low or non-existent. Parity, couple contraceptive use, husband’s serostatus and transmission risk perception remained significant predictors of safer conception practice on secondary analysis limited to couples that wanted to have babies (n = 150, Table 4).

## Discussion

Two-thirds of the HIV-infected women in this study were aware of the prospects of conceiving without infecting their partner or fetus. A similar proportion knew at least one method of safer conception. The most popular methods were ART, timed unprotected intercourse and a combination of the 2 methods. Nearly half of the respondents wanted more children within 3 years. Less than a quarter used contraceptives and an even lower proportion practiced safer conception. The practice of safer conception was associated with marital status, maternal occupation, the number of living children, husband’s serostatus, contraceptive use, and transmission risk perception.

The proportion of HIV-infected women who wanted more children was lower than the figure (75.8%) reported from Birnin Kudu in northern Nigeria.^27^ However, it was similar to the findings among HIV-infected women in Ethiopia (45.5%),^28^ but higher than among their counterparts in Uganda (35%).^29^ A multi-country study in 10 sub-Saharan African countries reported that fertility preferences and contraceptive behaviors of HIV-positive women were relatively similar across countries, where HIV-positive women were less likely to want more children and their partners more likely to use male condoms than HIV-negative women.^32^ Our figure was also lower than the proportions of HIV-infected women who were desirous of more children in the United States (50%) and the United Kingdom (75%).^33,34^ These differences could be related to variations in study population demographics, methods and currency of the studies, and cultural preferences.

The proportion of respondents aware of timed unprotected intercourse and artificial insemination as methods of safer conception were lower than that reported among their contemporaries in Uganda (51% and 53%, respectively).^29^ Further, the proportion of women aware of sperm washing was low in both studies (12.2% versus 15.0%), respectively.^35^ A qualitative study from Uganda also reported that while several clients had heard about safer conception methods, especially timed unprotected intercourse, only a few fully understood it. It was not surprising, therefore that, the couples felt that they were taking a gamble.^36^ Similarly, in Kenya, HIV-positive women mentioned safer conception methods including sperm washing, but could not provide details.^37^

The proportion of women who reported using the various methods of safer conception in the present study was higher than the baseline rates reported among their counterparts in Uganda, where 12% and 2% used timed unprotected intercourse and PrEP, respectively, while none had used manual self-insemination and sperm washing. Even after 24 months follow up, only 9% reported the use of PrEP, 3 male partners used manual self-insemination, while none used sperm washing.^29^ The situation was also similar among HIV-positive women in a study in South Africa.^30^ However, another South African study reported higher uptake of safer conception methods.^31^ This disparity could reflect the availability and familiarity with these methods among patients and healthcare providers. Considering the high premium accorded childbearing in sub-Saharan Africa, it is important to make low-technology, affordable safer conception methods accessible to couples that desire to procreate, satisfying the desire to bear children while reducing to a minimum the probability of mother-to-child transmission of HIV.^17^

The contraceptive prevalence among HIV-infected women was higher than that reported from Birnin Kudu, northern Nigeria (10%),^27^ and among the general populace in Nigeria (6.3%).^38^ Although, there was near universal support for the reproductive plans of HIV-infected women among partners and healthcare providers in our study, this was not the case elsewhere. For instance, more than 40% of women in Kenya reported being advised, primarily by healthcare providers and family members, to abstain from sex and terminate the pregnancy.^39^ Like others, women living with HIV are entitled to information and services that would guarantee safer sexual and reproductive choices.^40^ There is a need to integrate counselling on contraception, safer sex and reproduction for HIV positive couples into ART programs.

Our finding of an association between marital status, occupation and number of living children with safer conception practice is similar to reports from Uganda. In the Uganda study, however, additional factors such as perceived willingness to use safer conception methods, knowledge of respondent’s HIV status, HIV-seropositivity and equitable decision-making powers in relationships correlated with participants’ awareness and attitude towards safer conception methods.^35^ The role of marital status is not surprising, as pre-marital childbearing is abhorred in this setting. Therefore, it is unlikely that single HIV-infected women would have attempted to conceive, a likely reason for their limited knowledge of such methods. This group needs to be targeted with information before commencing procreation. Similarly, occupation, being a proxy for educational status is likely to influence health literacy and act as a cue for adopting safer practices. Women in skilled professions are likely to be better-educated and more likely to seek information and practice safer conception.^41^ However, the apparent paradox where use of safer conception was similar between civil servants and traders raises the possible role of affordability, as some safer conception methods could be expensive. As residents of the commercial center of northern Nigeria, traders in Kano tend to be wealthier than persons in other occupations. All HIV-positive women should nevertheless have access to such services to avoid transmission. Finally, children of HIV-positive women are more likely to survive if the mother adopted safer conception methods, hence the correlation between number of living children and ever practice of safer conception in this study.

Logistic model includes the following variables: age group, marital status, occupation, number of children, husband’s serostatus, couple contraceptive use, fertility desire and transmission risk perception.

It is not surprising that couple contraceptive use and risk perception were significantly associated with safer conception practice in our study. Both contraception use and the ability to perceive high transmission risk are indicative of a degree of level of knowledge of health-related issues. Couples who use contraception are likely to have had encounters with the health system and thereby become exposed to health education messages, which might include information on safer conception. The predictive role of husband’s serostatus on safer conception practice is also not unique. For instance, a qualitative study in Uganda demonstrated the importance of partner serostatus and couple communication dynamics on the uptake of safer conception methods.^42^ This finding highlights the influence of the male partner’s serostatus on the appropriateness of safer conception options and the dominant role of men in family decisions, especially in patriarchal settings. Our study findings underscore the importance of counselling HIV-affected couples on mutual disclosure, harmonization of fertility intentions and the adoption of safer conception methods that enable the achievement of couples’ reproductive goals at minimal risk to both parents and their offspring.

Our study has limitations. First, the study setting and participants may not be representative of the general population of women in northern Nigeria. Our respondents were a group of better-educated women, accessing ART services at a tertiary referral centre. They are likely to be more informed about safer conception, and to have higher contraceptive prevalence and lower fertility desires and intentions compared to their rural counterparts. Safer conception and family planning counselling and services are likely to be provided at such centres. Second, although individual interviews were conducted in private by trained interviewers from the same culture, social desirability bias cannot be ruled out. Responses could also differ in community-based surveys.

In conclusion, our findings highlight the mismatch between fertility desires/intentions, risk perception and safer conception practice in a low-resource setting. These findings are inimical to the goal of eliminating mother-to-child HIV transmission worldwide. Rather than being prescriptive, healthcare providers should pro-actively discuss fertility desires and intentions, the associated risks and safer conception options with their clients. This is often not the case in the study area and in South Africa where such conversations occur when client-initiated.^43^ It has been suggested that before any safer conception intervention, it is important, to the extent feasible, to ensure that the clients’ clinical state is favourable to a lower likelihood of transmission, specifically low viral load, high CD4+ cell count, and no AIDS-defining symptoms. Both partners should also be free of other sexually transmitted infections or should be receiving treatment, and should preferably be in a stable relationship.^44^ Where possible, fertility screening is also recommended, including semen analysis for HIV-infected men and the spinnbarkeit test for the woman to detect ovulation. Interventions should also be sensitive to the vulnerability of HIV-infected women, their unique psychosocial disposition and the considerable pressure they may face from male partners to get pregnant, even if they do not wish to.^17^ The prevention of unintended pregnancy is itself a major service component of prevention of mother-to-child transmission programs. Efforts should therefore be made to incorporate safer conception education in family planning programs, especially among women with HIV infection.

## Ethical issues

Ethical approval for this study was obtained from the AKTH Ethics Committee, approval number NHREC/21/08/2008/AKTH/EC/1576.

## Competing interests

Authors declare that they have no competing interests.

## Authors’ contributions

ZI, HSG, and AIO conceived and designed the study. AIO, AUG, and MB collected the data while ZI, MHA, and HSG analysed the data and prepared the draft manuscript. All authors reviewed and approved of the final manuscript.

## Authors’ affiliations

^1^Department of Community Medicine, Bayero University, Kano, Nigeria. ^2^Centre for Infectious Diseases Research, Bayero University, Kano, Nigeria. ^3^Department of Obstetrics and Gynecology, Bayero University, Kano, Nigeria. ^4^Department of Medicine, Bayero University, Kano, Nigeria. ^5^Department of Health Policy & Vanderbilt Institute for Global Health, Vanderbilt University Medical Center, Nashville, TN, USA.

## Key messages

**Implications for policy makers**

- Healthcare providers should pro-actively discuss fertility desires and intentions, and associated risks and safer conception options with their clients.
- Partner HIV testing and disclosure should be encouraged.
- Policies should prioritize building healthcare provider capacity to enhance safer conception counseling and service delivery.
- Couple contraceptive counseling and services should be provided as a component of programs for prevention of mother-to-child HIV transmission.

**Implications for public**

This paper provides recommendations for policy-makers and other stakeholders that would address the findings of high fertility desires and intentions, moderate risk perception and low uptake of safer conception practices among HIV-infected women in Kano, northern Nigeria. Well-designed and sustainable approaches to facilitating safer conception choices among such women will enhance the elimination of mother-to-child HIV transmission in Nigeria, the country with the highest burden of vertical HIV transmission in the world.
